# Molecular Hydrogen Maintains the Storage Quality of Chinese Chive through Improving Antioxidant Capacity

**DOI:** 10.3390/plants10061095

**Published:** 2021-05-29

**Authors:** Ke Jiang, Yong Kuang, Liying Feng, Yuhao Liu, Shu Wang, Hongmei Du, Wenbiao Shen

**Affiliations:** 1 Laboratory Center of Life Sciences, College of Life Sciences, Nanjing Agricultural University, Nanjing 210095, China; 2020816131@stu.njau.edu.cn (K.J.); 2020816132@stu.njau.edu.cn (Y.K.); 10119205@njau.edu.cn (L.F.); 2019816130@njau.edu.cn (Y.L.); 2019816129@njau.edu.cn (S.W.); 2 Center of Hydrogen Science, Shanghai Jiao Tong University, Shanghai 200240, China; 3 School of Design, Shanghai Jiao Tong University, Shanghai 200240, China; hmdu@sjtu.edu.cn

**Keywords:** Chinese chive, molecular hydrogen, storage quality, antioxidant capacity

## Abstract

Chinese chive usually becomes decayed after a short storage time, which was closely observed with the redox imbalance. To cope with this practical problem, in this report, molecular hydrogen (H_2_) was used to evaluate its influence in maintaining storage quality of Chinese chive, and the changes in antioxidant capacity were also analyzed. Chives were treated with 1%, 2%, or 3% H_2_, and with air as the control, and then were stored at 4 ± 1 °C. We observed that, compared with other treatment groups, the application of 3% H_2_ could significantly prolong the shelf life of Chinese chive, which was also confirmed by the obvious mitigation of decreased decay index, the loss ratio of weight, and the reduction in soluble protein content. Meanwhile, the decreasing tendency in total phenolic, flavonoid, and vitamin C contents was obviously impaired or slowed down by H_2_. Results of antioxidant capacity revealed that the accumulation of reactive oxygen species (ROS) and hydrogen peroxide (H_2_O_2_) was differentially alleviated, which positively matched with 2,2-Diphenyl-1-picrylhydrazyl (DPPH) scavenging activity and the improved activities of antioxidant enzymes, including superoxide dismutase (SOD), guaiacol peroxidase (POD), catalase (CAT), and ascorbate peroxidase (APX). Above results clearly suggest that postharvest molecular hydrogen application might be a potential useful approach to improve the storage quality of Chinese chive, which is partially achieved through the alleviation of oxidative damage happening during the storage periods. These findings also provide potential theoretical and practical significance for transportation and consumption of perishable vegetables.

## 1. Introduction

Molecular hydrogen (H_2_) has emerged as a potential therapeutic medical gas in medical treatment and clinical therapy because of its selectively antioxidant capability [1]. In the last several decades, there have been several reports indicating the presence of H_2_ in plants under normal or stressed conditions [2,3,4], although the detailed synthetic pathway(s) are not fully elucidated. Since 2012, evidence has been progressively obtained for the involvement of H_2_ in plant growth and development [5,6], as well as in defense responses in plants [7,8], when challenged with salinity [9], drought [10], osmotic stress [11,12], and heavy metal exposure [13]. On the other hand, the prolonged shelf life of fruits and flowers, including tomato [14], kiwifruit [15,16], cut rose [17,18], lisianthus [19], carnation [20], by means of molecular hydrogen and magnesium hydride [21], an effective H_2_-releasing material, has been discovered mainly in room temperature conditions. In most cases, the stimulation of antioxidant defense and the involvement of some other gaseous molecules, including nitric oxide [10] and hydrogen sulfide [22], as well as phytohormones [12] by exogenous H_2_ are proposed as the main mechanism in plants.

Chinese chive (*Allium tuberosum* Rottler ex Spreng.) is cultivated in China and other Asian countries as well as many European countries because this popular vegetable is rich in vitamins, fiber, and sulfur compounds with antibiotic properties [23,24]. Since Chinese chive is perishable and rapidly loses freshness during transportation and storage after harvesting, this vegetable is kept at ambient temperatures or usually stored at 4 °C [25]. Previous results revealed that leaf senescence of plants, including Chinese chive, is caused by reactive oxygen species (ROS) accumulation as well as the impaired ROS scavenging system, including the reduction in superoxide dismutase (SOD), guaiacol peroxidase (POD), and catalase (CAT) activities [26,27,28,29]. Although several approaches were proposed, including the application of the cytokinin compound [29] and storage under CO_2_-enriched atmospheric conditions [30], seeking more environmentally friendly and efficient methods is challenging for scientists and customers.

For the above purpose, this paper aimed to study whether or how H_2_ maintains the storage quality and extends the shelf life of fresh Chinese chive, delays senescence, and affects the ROS metabolism and antioxidant defense. These obtained findings have theoretical and practical significance, and also open a new window potentially for transportation and consumption of other perishable vegetables.

## 2. Results

### 2.1. Improvement of the Visual Quality of Chive during Storage in Response to Molecular Hydrogen

**Figure 1:**
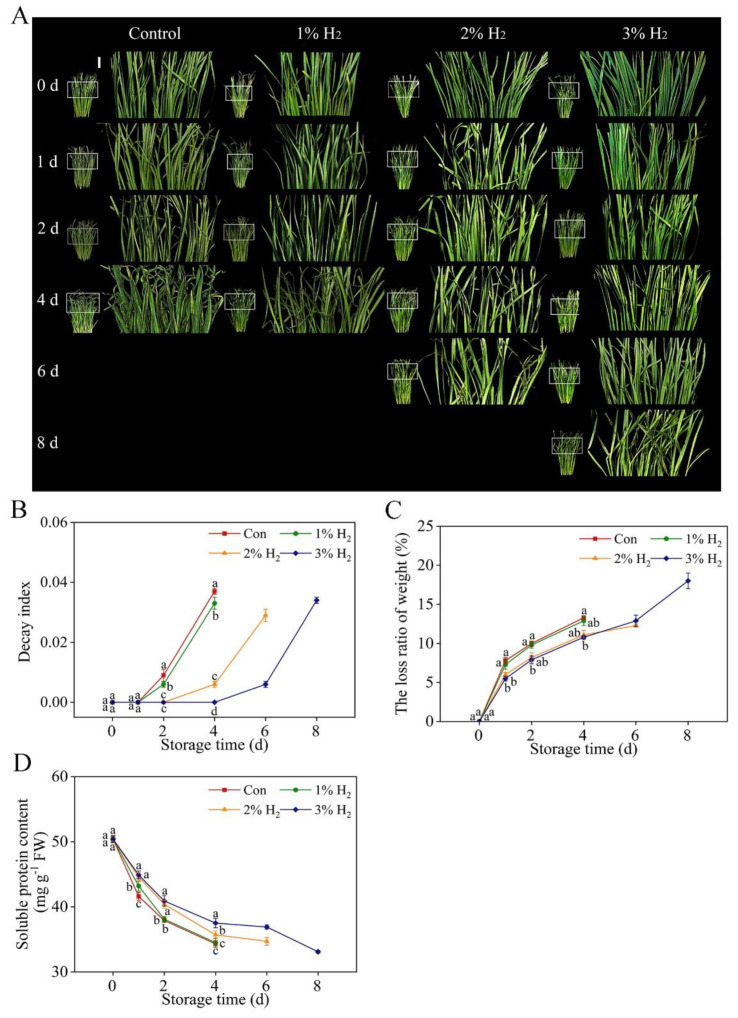
Effects of molecular hydrogen on shelf life of Chinese chive (**A**), decay index (**B**), the loss ratio of weight (**C**), and soluble protein content (**D**). Chinese chive was kept in air (control), 1% H_2_, 2% H_2_, and 3% H_2_ during storage at 4 ± 1 °C. Error bars represent the standard error (SE; *n* = 15 for decay index, *n* = 15 for the loss ratio of weight, *n* = 5 for soluble protein content). Bars with different letters for each storage time are significantly different (*p* < 0.05) according to Duncan’s multiple tests. Scale bar = 1 cm.

Compared to freshly harvested Chinese chives, which were green and free of decay, after storing at 4 °C for 4 d, chives deteriorated rapidly, showing yellowing as well as decaying. Unlike the changes in control (4.0 ± 0.4 d) and 1% H_2_ (4.0 ± 0.3 d), however, the application of 2% and 3% H_2_ could prolong the shelf life of chive, with 3% H_2_ showing the maximal responses (8.0 ± 0.3 d; Figure 1A).

During the time course of experiment, we also observed that visible signs of decay and wilting in chive were obviously delayed or slowed down by 2% and 3% H_2_ treatments, with respect to the control samples. Comparatively, weaker responses were observed in 1% H_2_-treated chive. Above results could be confirmed by the improvement of the decreased decay index and the loss ratio of weight (Figure 1B,C). For protein levels (Figure 1D), it was also observed that compared to the control group and 1% H_2_ treatment, the degradation of protein in chive leaves was differentially abolished by 2% and 3% H_2_ (in particular), the latter of which could be detected until 8 days of storage.

### 2.2. Changes of Total Phenolic and Flavonoid Contents

**Figure 2:**
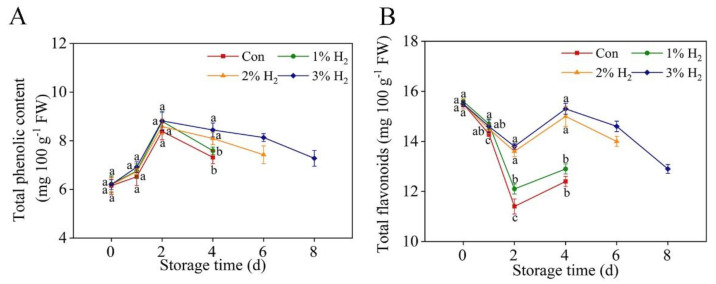
Time-dependent changes of total phenolic (**A**) and flavonoid (**B**) contents in response to molecular hydrogen. Chinese chive was stored at 4 ± 1 °C. Meanwhile, related parameters were analyzed. Means ± SE (*n* = 3 for phenolic and flavonoid contents, respectively) followed by different letters for each storage time indicate a statistical difference at *p* < 0.05.

Figure 2 shows the changes of the total phenolic and flavonoid contents in the presence or absence of H_2_ during postharvest period. For control and 1% H_2_-treated group, phenolic (Figure 2A) and flavonoid (Figure 2B) contents were increased or decreased during 2 days of storage, followed by decreasing or increasing until 4 days. In the presence of 2% H_2_ and 3% H_2_, however, changes of total phenolic and flavonoid contents after 2 days of storage were apparently slowed down or intensified, also keeping relatively higher levels until 6 days or 8 days of storage (especially for 3% H_2_ group).

### 2.3. H_2_ Slowed Down the Decreased Vitamin C

**Figure 3:**
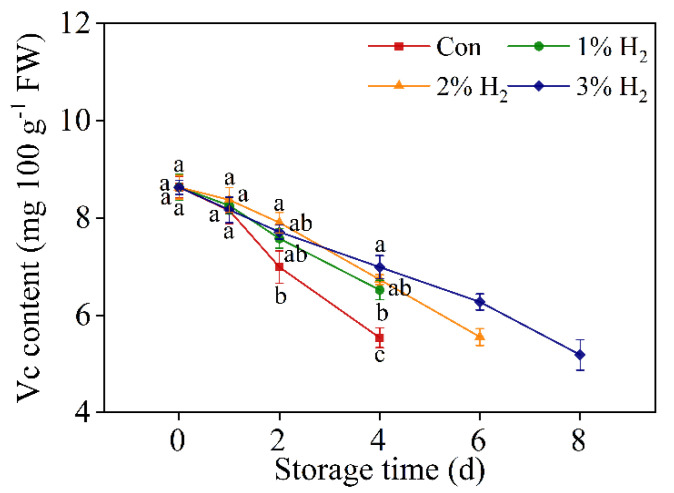
Time-dependent changes of vitamin C contents in response to molecular hydrogen. Chinese chive was stored at 4 ± 1 °C. Means ± SE (*n* = 3) followed by different letters for each storage time indicate a statistical difference at *p* < 0.05.

A time-course analysis of vitamin C levels during chive storage was analyzed by high-performance liquid chromatography (HPLC) after treatments in the presence or absence of H_2_ (Figure 3). As expected, in the control group, vitamin C contents were progressively decreased during the storage period, and were differentially abolished by 2% H_2_ and 3% H_2_ (in particular), lasting until 6 days or 8 days of storage. Comparatively, the application of 1% H_2_ brought about a weaker but also significant change in vitamin C level at 4 days of storage. Importantly, the above rescuing effects were approximately positively matched with the biological response of H_2_ in improving the visual storage quality of chive (Figure 1). Above results clearly suggest that the administration of molecular hydrogen can maintain vitamin C content in stored Chinese chive.

### 2.4. Redox Balance Was Reestablished by H_2_

**Figure 4:**
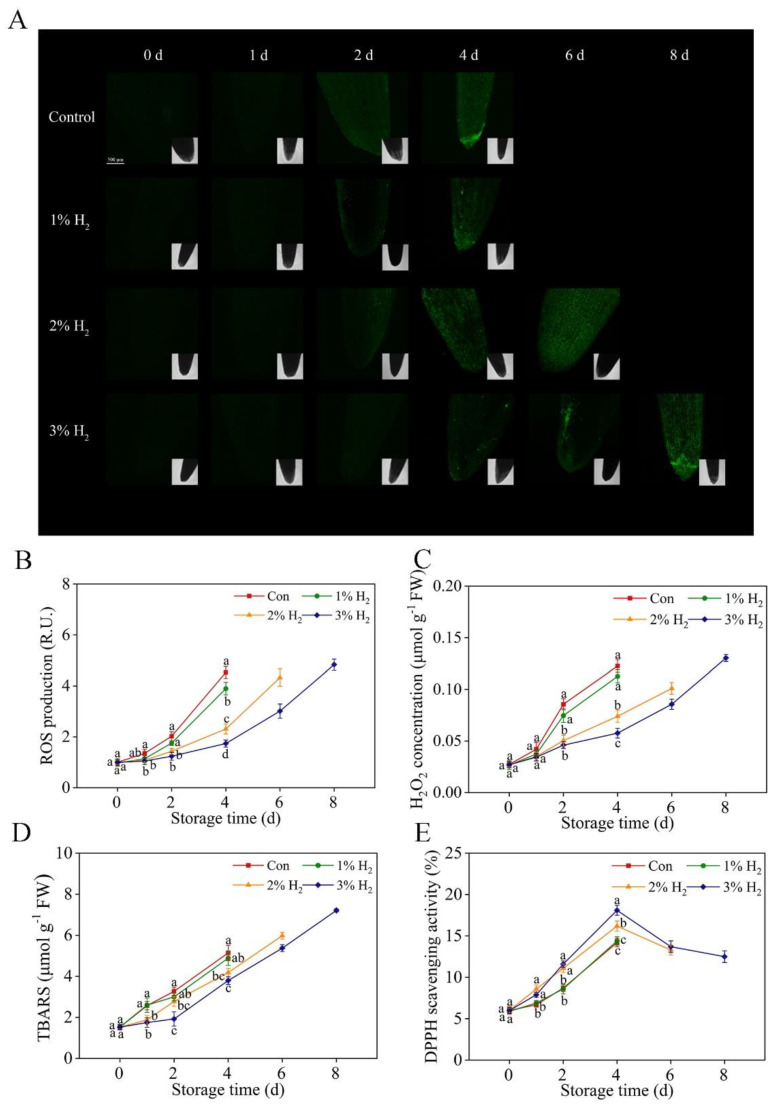
Redox balance was reestablished by molecular hydrogen. Chinese chive was stored at 4 ± 1 °C. The LSCM images of chive leaf apex loading with H_2_DCFDA (a ROS-specific fluorescent probe; (**A**)) were provided and the relative fluorescence was presented as values relative to Con at 0 day (**B**). R.U., relative units. Meanwhile, time-dependent changes in hydrogen peroxide (H_2_O_2_) levels (**C**), TBARS contents (**D**), and DPPH scavenging activity (**E**) of chives were determined. Means ± SE (*n* = 5 for LSCM imaging, *n* = 3 for H_2_O_2_ content, TBARS content, and DPPH scavenging activity, respectively) followed by different letters for each storage time indicate a statistical difference at *p* < 0.05. Scale bar = 500 μm.

It is well known that during storage and senescence, redox imbalance occurs, which could be evaluated as the accumulation of ROS and lipid damage [26,27]. To investigate the redox status, H_2_DCFDA (2′, 7′-Dichlorofluorescin diacetate), a ROS-specific fluorescent probe [10], was used to monitor ROS level in leave apex, followed by imaging by laser scanning confocal microscope (LSCM). As expected, during storage time, the fluorescence was progressively increased in the control group (until 4 days; Figure 4A,B), confirming the occurrence of redox imbalance during storage. By contrast, above fluorescence was apparently impaired or delayed until 6 days or 8 days of storage period by H_2_ in a dose-dependent fashion, with 3%, in particular, reflecting the possibility that redox balance in chives might be reestablished by H_2_.

To confirm the above deduction, both hydrogen peroxide (H_2_O_2_) and thiobarbituric acid-reactive substances (TBARS) contents (a reliable indicator of lipid damage) in leaf tissues were determined spectrophotometrically. Similar to the changes in above fluorescence, we also observed that the levels of H_2_O_2_ and TBARS contents during 4 days of storage period were apparently increased in a time-dependent fashion, which is partially blocked or delayed during 6 days or 8 days of storage period by different concentrations of H_2_ (except 1% H_2_ treatment), 3% in particular (Figure 4C,D). Consistently, results in the DPPH free radical scavenging assay demonstrated that H_2_-treated groups (3% in particular) were more effective than control group, with stronger antioxidant effects during the whole storage period.

### 2.5. Antioxidant Enzymatic Activates Were Stimulated in the Presence of H_2_

**Figure 5:**
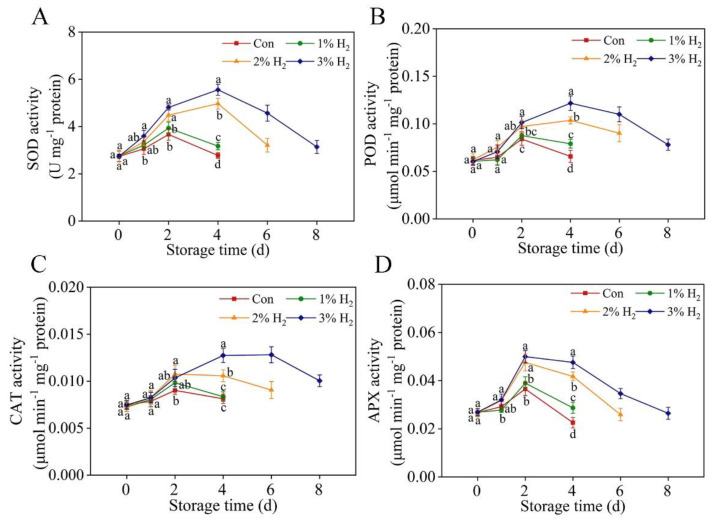
Time-dependent changes of SOD (**A**), POD (**B**), CAT (**C**), and APX (**D**) activities. Chinese chive was stored at 4 ± 1 °C. The enzymatic activities were expressed on a protein mass basis. Means ± SE (*n* = 5 for SOD, POD, CAT, and APX activities, respectively) followed by different letters for each storage time indicate a statistical difference at *p* < 0.05.

Next, to investigate whether the alleviation of oxidative damage during the storage period was causally caused by the increased antioxidant defense, activities of representative antioxidant enzymes, including SOD, POD, CAT, and ascorbate peroxidase (APX), were determined. Results shown in Figure 5 reveal that during 4 days of storage time, activities of the above four antioxidant enzymes in control sample displayed increasing tendencies (until 2 days), followed by decreasing during the rest of the storage period. Similar tendencies were observed in 1% H_2_-treated group. By contrast, total activities of these enzymes were intensified until 4 days (SOD, POD, and CAT) or 2 days (APX) of storage periods, and slowed down thereafter until 8 days in other H_2_ groups, and these effects were maximal in the presence of 3% H_2_.

### 2.6. Changes in Reduced Glutathione (GSH) and Glutathione Reductase (GR) Activity

**Figure 6:**
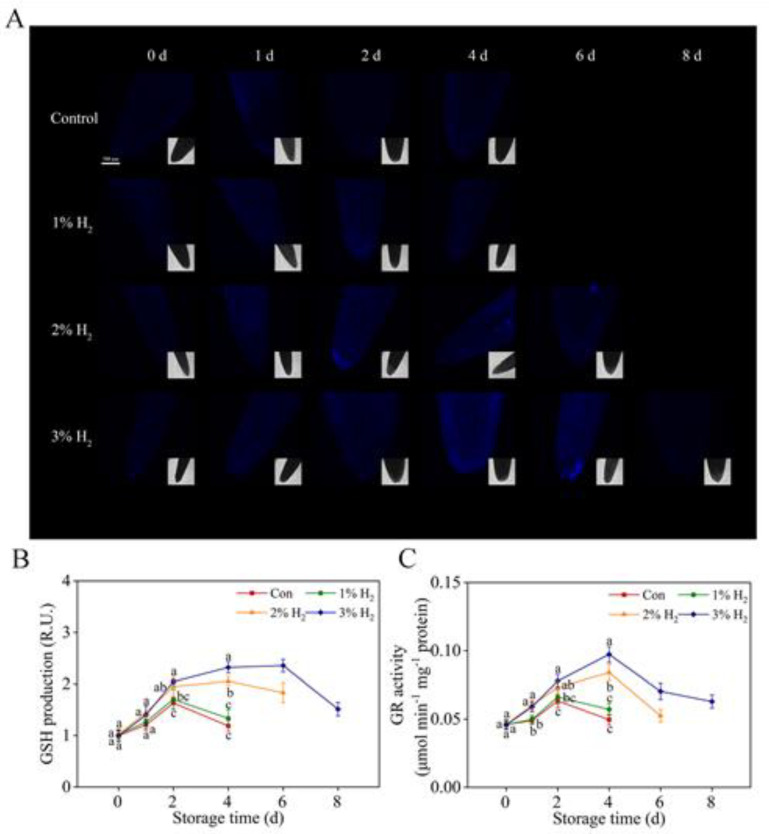
Changes in reduced glutathione (GSH) content and GR activity. The LSCM images of monochlorobimane (MCB)-dependent fluorescence in chive leaf apex were used to represent reduced GSH contents (**A**) and the relative fluorescence was presented as values relative to Con at 0 day (**B**). Meanwhile, time-dependent changes in GR activity (**C**) were determined. Means ± SE (*n* = 5 for LSCM imaging and GR activity, respectively) followed by different letters for each storage time indicate a statistical difference at *p* <0.05. Scale bar = 500 μm.

To determine whether the above molecular hydrogen responses resulted from influencing non-enzyme antioxidant substance and its metabolism, changes in reduced glutathione (GSH) content and glutathione reductase (GR: one of the synthetic enzymes for GSH metabolism) activity were also determined. For endogenous GSH to be tracked in situ, a commercial specific fluorescent probe monochlorobimane (MCB) was used (Figure 6A,B). As expected, in control and 1% H_2_-treated group, the MCB-dependent fluorescence was increased during 2 days of storage period, followed by a decrease until 4 days. Further results illustrated that above fluorescence was obviously impaired or delayed until 6 days or 8 days of storage period by 2% and 3% (in particular) H_2_ groups, reflecting the possibility that non-enzyme antioxidant substance in chives might also be influenced by H_2_. Meanwhile, we observed that activities of GR displayed similar tendencies (Figure 6C).

## 3. Discussion

Hydrogen-based agriculture, called hydrogen agriculture, belongs to a new low-carbon economy, which mainly refers to the application of H_2_ or related storage/releasing materials for improving the production and quality of crop, forest, livestock, aquatic, and other related agricultural products during, before, and/or post-harvesting periods [5,6,8]. Here, we showed that postharvest molecular hydrogen application can maintain the storage quality of Chinese chive through improving antioxidant capacity.

Senescence is a major limiting factor in keeping chives fresh after harvesting [30]. It is well documented that both decay and weight loss are two important indexes which are closely associated with the commercial value of fruits and vegetables [31], especially for Chinese chive, which is normally only kept less than one week in open market or supermarket, even storing at 4 °C. In this report, we found that the application of 3% H_2_ treatment can prolong the shelf life of chives, from 4 days of control treatment to 8 days (Figure 1A). It is a new finding. This result was further closely matched with the alleviation of the reduction in decay index (Figure 1B), the loss ratio of weight (Figure 1C), as well as the mitigation of soluble protein degradation in chives when 3% H_2_ was applied (Figure 1D). Comparatively, changes in total phenolic and flavonoid contents (Figure 2) as well as vitamin C levels (Figure 3) during the storage period of chives in the presence of 3% H_2_ displayed similar tendencies, and the rescuing effect in decreased vitamin C content by molecular hydrogen was previously discovered in tomato fruits after harvesting [14]. Above results clearly indicated that postharvest molecular hydrogen application could prevent the loss of nutriments during vegetable storage.

Besides vitamin C, exogenous application with hydrogen-rich water could prevent nitrite accumulation and further delay senescence of tomato fruit during storage [14]. Our study here showed similar ameliorative physiological phenotypes in Chinese chive, but for this case, we used hydrogen gas instead. Since some vegetables and fruits are prone to be perishable during storage when contacted with liquid solutions [32], our results are significant for both fundamental and applied agriculture.

Subsequently, we found that H_2_ improved the preservation ability of Chinese chive by reestablishing redox homeostasis. It is well known that during storage, the occurrence of redox imbalance, caused by the accumulation of ROS, is one of the most important factors accelerating senescence during the storage of vegetables and fruits [33]. Normally, ROS accumulation is characterized by fast superoxide anion radical productive rate and therefore more H_2_O_2_ content, thus resulting in membrane lipid peroxidation [34,35], which is expressed as TBARS content. In our experiments, the accumulation of ROS and H_2_O_2_, and thereafter lipid peroxidation in leaves of chives were less pronounced in the presence of 3% H_2_, especially under the similar time points, compared with the control group (Figure 4A–D), indicating that H_2_ reduced the damage of excessive ROS in chives. Above results might be explained by changes in DPPH scavenging activity conferred by molecular hydrogen (Figure 4E). Redox homeostasis was therefore reestablished to some extent.

Previously, the improvement of antioxidant defense by H_2_ was proposed as the main mechanism in plant response against different stresses [9,10,12]. The present work further indicated that 3% H_2_ treatment significantly increased SOD activity in chives (Figure 5A). Thus, the lower production of superoxide anion radical might happen. The reduction of H_2_O_2_ content, one of the products of SOD, was also observed in the presence of 3% H_2_ (Figure 4C). Since the scavenging of H_2_O_2_ is achieved by CAT, POD, and APX [13,19], activities of the above three enzymes were further analyzed. As expected, this study clearly showed that 3% H_2_ treatment obviously increased their activities (Figure 5B–D), all of which further resulted in lower levels of H_2_O_2_ in chives (Figure 4C).

It is well known that both antioxidant enzymes and non-enzymatic antioxidant substances responsible for reestablishing redox homeostasis are responsible for delaying senescence in plants [18,20,36]. Recent results showed that GSH-related characters as well as antioxidant defense were closely related to the function of molecular hydrogen in delaying the pericarp browning of litchi [37]. Our further results discovered that, consistent with the changes in redox homeostasis (Figure 4), the administration of 3% H_2_ could enhance GSH content and increase GR activity (Figure 6), one of the synthetic enzymes for GSH metabolism [38]. Importantly, these changes also matched with the beneficial phenotypes triggered by molecular hydrogen (Figure 1).

Overall, these results clearly showed that molecular hydrogen was an ideal treatment for Chinese chive storage, since 3% H_2_ effectively maintained storage quality compared with the control group at 4 °C storage, which could be attributed to increased activities of antioxidant enzymes and GSH content as well as the reduced ROS accumulation in Chinese chive. Our findings therefore provide a more practical approach for transportation and consumption of some perishable vegetables and fruits.

## 4. Materials and Methods

### 4.1. Plant Materials and Treatments

Fresh Chinese chive (*Allium tuberosum* Rottler ex Spreng.) without defects, diseases and physical damage was purchased from the Suguo supermarket (Nanjing, Jiangsu Province, China; chives’ place of origin: Lishui, Nanjing) and was quickly transferred to the laboratory. Afterwards, chives with uniform color and size and no tendency for withering and yellowing were selected for the subsequent experiments.

During the whole experiment, Chinese chive was stored in sealed plastic containers (1.5 L, Lock & Lock) containing air (control), and 1%, 2%, or 3% H_2_. Through calculation, a certain volume of air was extracted through the injection port on each container, and then the molecular hydrogen produced by the H_2_ generator was immediately injected into the container according to the corresponding volume, so as to achieve the experimental requirements. All treatment gas was renewed daily, and the plastic containers were kept in refrigerator at 4 ± 1 °C with a relative humidity (RH) of 70–75% in darkness. The sample tissue used for further analysis was the leaves of chives.

### 4.2. Preparation of Hydrogen Gas

Purified hydrogen gas (H_2_, 99.99% (*v/v*)) was generated from H_2_ generator (SHC-300; Saikesaisi Hydrogen Energy Co., Ltd., Shandong, China).

### 4.3. Determination of Decay Index and the Loss Ratio of Weight

According to the previous methods [29,39,40], the decay index of Chinese chive was determined based on the area percentage of the tissue affected by any decay, followed by scoring on a 1 to 5 scale; a score of 3 represented that the product was usable but not salable. For the measurement of the loss ratio of weight, the fresh weight (FW)of Chinese chive in each treatment was weighed at a fixed time every day [29,39].

### 4.4. Determination of Total Phenolic, Total Flavonoid

According to the previous method, total phenolic content in Chinese chive was measured by the Folin–Ciocalteu method [41] with a minor modification. First, 0.5 g of tissues was homogenized in liquid nitrogen and extracted by 80% acetone for 30 min. After centrifugation, 1 mL of the supernatant was incubated with 2 mL of undiluted Folin–Ciocalteu reagent for 2 min, and then 10% (*w/v*) Na_2_CO_3_ solution was added. After incubation for 1 h at 50 °C, the absorbance of the mixture was measured at 765 nm by using spectrophotometry (UV-2802 spectrophotometer, Shanghai Unico Instruments Co., Ltd., Shanghai, China). Total phenolic content was calculated from a standard curve for gallic acid, and expressed as milligrams of gallic acid per 100 g of fresh weight of sample.

Following the previous methods [41,42], the content of total flavonoid was determined by spectrophotometry. Chive tissues (0.1 g) were extracted with acetone/water/acetic acid (70:29.5:0.5, *v/v/v*) solution for 30 min. Afterwards, 4 mL of distilled water and 0.3 mL of 5% NaNO_2_ (*w/v*) were added to 1 mL of the extract and then incubated for 5 min. Then, 0.3 mL of 10% AlCl_3_ (*w/v*) and 2 mL of 1 mol L^−1^ NaOH were added to the mixture separately. After reaction in the dark for 15 min and centrifugation for 5 min, the absorbance was determined at 510 nm on a spectrophotometer. A standard curve was obtained by adding a variable amount of catechin. Total flavonoid content was expressed as milligrams of catechin per 100 g of FW.

### 4.5. Laser Scanning Confocal Microscope

According to the previous method [43], the ROS in chives were determined using a Zeiss LSM 800 confocal microscope (Carl Zeiss, Oberkochen, Germany). Briefly, the leaf apex of Chinese chive was incubated with 25 μM 2′, 7′-dichlorofluorescin diacetate (H_2_DCFDA; Sigma-Aldrich, Saint Louis, America, http://www.sigmaaldrich.com (accessed on 25 April 2021)) for 20 min in the dark. After washing with HEPES/NaOH buffer (pH 7.5) three times, the sample was detected immediately by confocal microscope. Detection was performed by λ (excitation) = 488 nm and λ (emission) = 500–530 nm. The relative fluorescence was expressed as values relative to the control group at 0 day.

The reduced GSH content in chives was estimated following the previous method [44]. The leaf apex of Chinese chive was incubated with 50 μM monochlorobimane (MCB; Sigma-Aldrich, Saint Louis, America, http://www.sigmaaldrich.com (accessed on 25 April 2021)) for 20 min in the dark and was washed with HEPES buffer (pH 7.5) three times. Subsequently, the sample was detected immediately by a Zeiss LSM 800 confocal microscope (Carl Zeiss, Oberkochen, Germany; emission at 461 nm, excitation at 380 nm, respectively). The relative fluorescence was presented as values relative to Con at 0 day.

### 4.6. Determination of Hydrogen Peroxide (H_2_O_2_) Content

The H_2_O_2_ content was measured by spectrophotometry according to the previous method [45]. Chinese chive tissues (0.5 g) were ground with 2 mL of 0.2 mol L^−1^ HClO_4_ on ice. After centrifugation at 12,000× *g* for 15 min at 4 °C, 0.5 mL of the supernatant was mixed with 50 mmol L^−1^ H_2_SO_4_, 0.5 mmol L^−1^ ammonium ferrous sulfate, 200 mmol L^−1^ sorbitol, and 0.2 mmol L^−1^ xylenol orange. Afterwards, the assay reagent was incubated at 45 °C for 30 min, and the absorbance was measured at 560 nm. A standard curve was obtained by adding different amounts of H_2_O_2_.

### 4.7. Assay of Thiobarbituric Acid Reactive Substances (TBARS) Content

The content of TBARS in Chinese chive was determined by a spectrophotometer as previously described, with a minor modification [46]. The sample tissues (0.2 g) were homogenized with 2 mL of 0.1% (*w/v*) trichloroacetic acid (TCA) on ice. After centrifugation at 12,000× *g* for 15 min, 0.5 mL of the supernatant was added to 1.5 mL of thiobarbituric acid (TBA). After the assay reagent was incubated at 90 °C for 20 min, the absorbance of the sample was measured at wavelengths of 532 nm, 600 nm, and 450 nm. TBARS content was expressed as µmol g^−1^ FW.

### 4.8. Determination of 2,2-Diphenyl-1-Picrylhydrazyl Radical (DPPH) Scavenging Activity

DPPH scavenging activity was determined according to previous method [15]. Briefly, the solution containing 10 µL of methanol extract and 3 mL of 0.1 mmol L^−1^ DPPH-methanol solution was incubated for 30 min at 25 °C in the dark. The decrease of the absorbance was measured by using a spectrophotometer at 517 nm, and blanks contained methanol instead of DPPH solution.

### 4.9. Determination of Vitamin C Content

The content of vitamin C in Chinese chive was estimated by using the previous methods [14,47]. The tissues of Chinese chive were derivatized with 1,2-*o*-phenylenediamine after extracting with trichloroacetic acid solution. The vitamin C content was determined by HPLC (D-2000, Hitachi, Ltd., Tokyo, Japan; excitation at 350 nm, emission at 430 nm, respectively). Quantification of vitamin C content was carried out by external calibration with *L*-ascorbic acid and expressed as mg 100 g^−1^ FW.

### 4.10. Assay of Antioxidant Enzyme Activity

In order to determine the activities of SOD, POD, CAT, APX, and GR, fresh tissue samples (0.2 g) from Chinese chive were homogenized in 3 mL of 50 mmol L^−1^ phosphate buffer (pH 7.0) [19]. SOD activity was determined by detecting the inhibition of photochemical reduction of nitro blue tetrazolium (NBT) at 560 nm according to the previous methods [19,48], and one enzyme unit (U) was considered to be the amount of enzyme corresponding to 50% inhibition of NBT reduction. POD activity was determined by following the oxidation of guaiacol at 470 nm [13]. CAT activity was estimated by detecting the reduction of H_2_O_2_ at 240 nm [48]. APX activity was assayed by monitoring the decrease at 290 nm after adding 1 mmol L^−1^ ascorbic acid [13]. GR activity was determined by monitoring the oxidation of nicotinamide adenine dinucleotide phosphate at 340 nm [49]. The enzyme activity was expressed on a protein mass basis, and the protein concentration of Chinese chive was determined by BCA (bicinchoninic acid) Protein Assay Kit (TaKaRa Bio Inc., Dalian, China).

### 4.11. Experimental Design

Following the previous reports [19,50] with some minor modifications, all experiments were arranged in a randomized complete block design. The experiment was carried out 3 times with triplicates per experiment, and each replicate included 15 Chinese chives. In order to determine decay index and the loss ratio of weight, 15 chives were selected for determination each time, and the total number of three repeated chives was 45 (15 × 3), and the representative phenotypes were photographed. Five chives per replicate were selected for the evaluation of LSCM imaging, soluble protein content, and enzyme activities, and the total of three repeated chives was 15 (5 × 3). For other parameters, including total phenolic and flavonoid contents, vitamin C content, H_2_O_2_ concentration, TBARS content, and DPPH scavenging activity, three chives were selected for each repetition, and the total of the three repetitions was 9 (3 × 3). All samples used for the determination were taken from healthy tissues of the samples.

### 4.12. Statistical Analysis

All values in this study were expressed as the means ± standard error (SE) from three independent experiments with three biological replicates for each. Data analysis was performed by using SPSS 16.0 software (SPSS Inc., Chicago, IL, USA), and one-way analysis of variance (ANOVA) was used to analyze differences among treatments according to Duncan’s multiple range test, and *p* < 0.05 as significant.

## Data Availability

All data reported here is available from the authors upon request.

## Abbreviations

APX	ascorbate peroxidase
ANOVA	one-way analysis of variance
BCA	bicinchoninic acid
CAT	catalase
Con	control
DPPH	2,2-Diphenyl-1-picrylhydrazyl
FW	fresh weight
GSH	glutathione
GR	glutathione reductase
H_2_	molecular hydrogen
H_2_DCFDA	2′, 7′-Dichlorofluorescin diacetate
HPLC	high-performance liquid chromatography
H_2_O_2_	hydrogen peroxide
LSCM	laser scanning confocal microscope
MCB	monochlorobimane
NBT	nitro blue tetrazolium
POD	guaiacol peroxidase
RH	relative humidity
ROS	reactive oxygen species
R.U.	relative units
SE	standard error
SOD	superoxide dismutase
TBARS	thiobarbituric acid reactive substances
